# Medical and Physical Researches, or Original Memoirs in Medicine, Surgery, Physiology, Geology, Zoology, and Comparative Anatomy

**Published:** 1839

**Authors:** 


**Medical and Physical Researches, or Original Memoirs in Medicine, Surgery, Physiology, Geology, Zoology, and Comparative Anatomy. Illustrated with Plates, containing 160 Figures. By *R. Harlan*, M.D. F.R.S. Lond. &c. &c. &c. Philadelphia. Royal octavo, pp. 653. 1835.**

The volume before us is chiefly devoted to the collateral sciences of geology, zoology, and comparative anatomy. The essays which relate to them are replete with information, and will repay perusal. But they are not adapted to our pages, and we must necessarily pass them by. To do so without complimenting our transatlantic author, on his zeal and acquaintance with the subjects he discusses, would be an act of positive injustice.

The medical or surgical papers are found in a narrow corner of the volume. They are unconnected with each other, and many are of too general a cast to suit our purpose. We must content ourselves with noticing a few briefly, apologizing to Dr. Harlan for the small space that we can spare.

### 1. *Case of Aneurysm of the Pulmonary Artery.*

Cases of this disease are sufficiently rare, to render what do occur objects of curiosity.

*Case.* In September, 1814, Dr. Harlan assisted Dr. Parrish in the examination of the body of Capt. Man—a middle-aged person of robust constitution. His disease had never confined him to his house, and the day preceding his death, he called on Dr. P. and complained of dyspnœa, and pain in the back of his neck. His symptoms were referred to stricture of the œsophagus, and Professor Wistar, who was consulted, advised the use of the probang; the patient deferred this operation, and during the same night was attacked with increased difficulty of breathing, and exacerbation of all his symptoms. A bleeder being sent for, he lost some blood, with temporary relief—but in a short time after, he expired in convulsions.

Autopsy, about eighteen hours after death, very unexpectedly developed an aneurysm of the pulmonary artery—the sac of which had extended so far back as to press upon the œsophagus, and produced symptoms of stricture in that part. There was considerable effusion in the lungs, and a lump of coagulated blood in the trachea, near the glottis—which most probably occasioned his sudden death. The effusion was occasioned by the rupture of the aneurysmal sack, which was adhering to the air-cells of the lungs.

Dr. Harlan justly observes, that it was fortunate the probang was not employed.

### 2. *Treatment of Carbuncle with the Caustic Alkali.*

In this country, we always make use of crucial incisions, and with what success everybody knows. But Dr. Harlan recommends the caustic alkali as less painful. We confess that we doubt its comparability with incisions. But let the Doctor speak for himself.

Applied in the earlier stages of the disease, it immediately arrests the specific action, and establishes healthy suppuration, whilst in the ulcerative stage, it very soon changes the morbid action, and at a still more progressed stage, it separates the sloughs, and produces a healthy sore.

The caustic alkali should be used liberally, and followed immediately by a bread and milk poultice, for which, the application of a mixture of basilicon ointment and spirits of turpentine is occasionally substituted, in order to hasten the separation of the sloughs; this, with the necessary attention to the constitutional symptoms, the treatment of which is to be modified according to the state of the system, is all that is requisite to the cure of carbuncle.

### 3. *Case of Prolific Uterus and Barren Mammæ.*

In October, 1820, Dr. Harlan's services were requested by Mary Ann Gready, aged 30, native of the North of Ireland. She is the youngest daughter of twenty-two children, thirteen of whom attained maturity; her mother never secreted a drop of milk wherewith to nourish her infants. Mary Ann, the subject of the present observations, was delivered by Dr. H. of her thirteenth child, a perfectly-formed female. Out of these thirteen progeny, two were aborted, from accidents; two were premature; four are living; and five died from disease. On the third day after parturition, the mammæ are observed to swell a little, and to become slightly painful; on the fourth day they subside again. About the third week after parturition, a stomachic affection supervenes, such as pain, eructation, nausea, vomiting, and other dyspeptic symptoms, which continue until she again becomes pregnant, which has always occurred in five months after delivery. No uncommon appearance of the mammæ is perceptible—they being adequately developed.

### 4. *Dr. Harlan's Experience of the Sugar of Lead in Dysentery.*

"In the Summer of 1820, while prescribing in the Dispensary of this city, I employed the saccharum saturni in many cases of dysentery, and the result of my experience has convinced me that it is a safe and efficacious remedy in this disease. I have found it, in the majority of cases, to check the bloody stools, to allay intestinal irritation, and to relieve, in a very prompt manner, tormina and tenesmus. It will be observed that I did not generally prescribe the sugar of lead by itself; and it may, therefore, be inferred, that the remedies with which it was combined, had the principal share in the remediate effects of my practice. This may be so, in some of the cases, but I am, notwithstanding, fully satisfied that the sugar of lead was of much service in the prescriptions ordered; and this I infer from my having uniformly derived more advantage from my prescriptions when the sugar of lead was added, than when it was omitted. In some instances it was administered alone, or combined only with small doses of opium." 567.

We subjoin one case in illustration:—

*Case.*—Sept. 12. James Roberts. This was a very severe case; the tormina and tenesmus were exceedingly great. The patient discharged considerable quantity of blood. Dr. H. saw him within the first twenty-four hours, and immediately ordered:—

Sacch. Saturn. gr. xviii.

Pulv. Opii. gr. vi.

M. ft. pulv. vi. One to be taken three times daily.

By the use of this medicine alone this patient was perfectly cured in three days. The tenesmus, tormina, and bloody discharges were promptly relieved, and the cure was permanent.

Dr. Harlan adds:—

"It appears from the works of Jackson, and Moseley, that sugar of lead has been used, both in acute and chronic dysentery, in the West Indies. This I was not aware of until about to draw up these remarks. I believe, however, that this remedy was not used, in such large doses, with similar views, with the same results, and to the same effect, previous to my having used it so very extensively and beneficially in the obstinate epidemic of 1820. I do not, indeed, know that the sugar of lead was ever used at all in the acute dysentery, in this city, previous to the summer of 1820. Whatever may be the origin of this practice, however, I am fully satisfied, that the sugar of lead, properly administered, is calculated to prove extensively useful in dysenteric affections." 570.

5. *Observations on Colica Pictonum, and on the Use of the Acetate of Lead in that Disease.*

Dr. Harlan has had, for a number of years, the professional charge of the labourers attached to one of the most extensive white-lead manufactories and chemical laboratories in the United States. His opportunities of becoming acquainted with the disease and its treatment have been, therefore, far from inconsiderable.

The men employed are for the most part of robust constitutions, and chiefly natives of Ireland and Germany. A certain degree of susceptibility or predisposition seems a necessary antecedent to the attack. Drunkenness, or habitual dissipation of any kind, renders the system peculiarly liable to the disease, and frequently leads to a fatal issue. Sudden transitions of temperature, together with exposure to cold and moisture, are predisposing causes. The cases were most common late in the Spring and early in the Autumn.

*Danger of particular parts of the Process.*—Of the youths who constantly work at rolling the sheets of lead, in the melting room, only one has been affected with *lead disease*. A boy, about twelve years of age, was attacked at night with paralysis of all his extremities, and with pain in the abdomen. He was treated with mercury, cathartics, &c. He recovered—returned to work—and again relapsed in three or four days. He finally recovered, with the usual remedies, and was discharged from the manufactory.

The different stages of manipulation in the manufacture of white-lead, render the labourer obnoxious to these affections, and also give rise to some peculiarities in the attack and in the symptoms. The most dangerous operation consists in the packing of the kiln-dried white-lead into barrels, a process performed by *negroes*, who are employed for the purpose at short periods, and at considerable intervals. Among these several cases have occurred.

Those hands employed in melting the metal in the casting-room, are more liable to be attacked with apoplectic or epileptic convulsions, without pain in the bowels during the first stage; and such cases are more apt to terminate in *paralysis*.

Frequent opportunities have occurred to demonstrate that man is by no means the only animal subject to this disorder. Dogs, cats, cows, and chickens, not unfrequently die with this affection. In the latter, "post mortem" examinations have displayed mortification of the intestines. Symptoms of cerebral derangement have manifested themselves in the feathered tribe, when labouring under the deleterious effects of white-lead. After a few moments of cataleptic abstraction, they have been observed to dash their heads against the wall, or any other resisting object. Instances of the disease have been observed in pigeons, which frequent the ordure bank in the lead factory yard. Immediately preceding death, they are occasionally observed to ascend into the air to an immense height, and fall dead to the earth. On dissection, mortification of the intestines is detected.

*Modes of Attack.*—Dr. Harlan troubles himself with no hypothesis on the intimate mode of action of the lead. He believes that it may be absorbed into the system either by the stomach, lungs, or skin.

Yet, he adds, "it does by no means invariably produce colic, pain in the bowels, or constipation, as an early symptom. On the contrary, I have frequently observed that some of the most obstinate and severe cases of 'lead sickness,' are ushered in by a diarrhœa, sometimes continued for two days, but which is mostly checked by proper remedies in a few hours. In other cases, the patient is attacked, generally at night, with symptoms which I have designated as *apoplectic*, from the suddenness of the attack, the stertorous breathing, and insensibility, generally attendant; but at the same time, it must be remembered, that there exists a state of the system very different from that which prevails in apoplexy. Together with the symptoms above stated, the patient is cold, particularly his extremities; his countenance, in place of being suffused, is pallid; and convulsions of the whole frame frequently occur. This order of things continues from a few hours to three or four days. In these cases, as might have been anticipated, the patients do not bear general bleeding. The best practice consists in the administration of calomel and opium, notwithstanding the constipation of the bowels and determination to the head, resorting, at the same time, to the early use of cathartics. These remedies, assisted by the application of cups to the head, and a blister to the back of the neck, with rubefacients to the lower extremities, will generally prove successful in curing the disorder, or in transferring it to the bowels. I have witnessed one case of this description treated differently, which terminated in the death of the patient." 574.

But by far the greater number of patients afflicted with "lead sickness," display the symptoms of old-fashioned colica pictonum, or "dry belly-ache;" in which case we find them labouring under excruciating griping pains in the region of the umbilicus; the skin of the abdomen is thrown into folds or knots; obstinate constipation prevailing, with febrile excitement; the tongue being sometimes white, at others covered with a thick brown fur. The pain is principally spasmodic, occurring at intervals of different durations, and is seldom entirely intermitting.

*Treatment.*—Dr. Harlan informs us that, after trying various remedies, his practice has long been settled.

"We will take, for example, a violent case, characterized by the above detailed symptoms. It is my practice to bleed from the arm; and in some cases cups are applied to the abdomen, or a mustard plaster, followed by an epispastic over the inflamed skin. But, at all events, I lose no time in administering calomel and opium, in the proportion of ten grains of the former to two or three of the latter; and, if there be no nausea present, two grains of ipecacuanha are added. The whole powder is repeated every two or three hours, until pain is relieved, or ptyalism is induced; it is then omitted, and one or other of the following cathartics given. ℞. sal. eps. ℥j., magnes. ustæ. ℥ss.; mix them in a pint of water, and take from a wine-glassful to a teacupful every two hours. Or, ℞. rad. rhei. contus. ℥ss., fol. sennæ ℥j., sem. cardam. ʒij.; decoct in one pint of water. The dose is one wine-glassful every two hours, until the bowels are evacuated.

At this stage of the disease, patients will require large doses of the cathartic; and, if administered earlier in the case, or previous to the free administration of mercury and opiates, no quantity that the stomach is capable of containing can be made to produce a cathartic effect on the bowels. I have frequently witnessed the whole quantity of both the above prescriptions taken, together with the addition of castor oil, without any effect. As the excitement of ptyalism is of vital importance in violent cases, the blisters on the abdomen are to be dressed with mercurial ointment, and mercurial frictions, to a greater or less extent, are to be resorted to; especially if any torpor or insusceptibility in this respect is observed in the patient.

As valuable adjuvants in such obstinate constipations, the use of turpentine injections, or injections of molasses and water, with half an ounce of table-salt, is next to be resorted to. In some cases, where the heat of the abdomen was very great, attended with soreness on pressure, anxiety, and restlessness, with other symptoms denoting approaching enteritis, the most decided advantages have been obtained by the use of large and frequently repeated injections of cold pump water. In this manner heat is abstracted and inflammation prevented or arrested. Affusions of cold water on the lower extremities have also been resorted to with success in similar constipated cases. This treatment, under ordinary circumstances, will seldom fail of success. One patient recovered after having displayed every character of the hippocratic countenance: a state of things, in the present instance, induced by gastric sympathies; the patient, previous to being relieved, having ejected from the stomach a large quantity of matter, resembling thick green paint." 576.

The most violent and unmanageable cases are those attended with extreme irritability of stomach, which rejects every thing. Dr. H. has employed laudanum, peppermint, calomel and opium, and oil of turpentine, together with the external application of spices and rubefacients. When all these have failed, he has succeeded by means of a remedy which might not, à priori, have been thought advisable, the powdered acetate of lead. The advantage which he derived from it in dysentery led him to employ it in the "colic." He resorts to the following formula:—

℞. Cal. ppt. gr. v., pulv. opii. gr. ij., pulv. sacch. sat. gr. iij.—m. ft. pulv.; to be repeated every two hours until relief is obtained; which is generally the case after two or three powders have been taken. Injections of saccharum saturni, with opium in solution, have also been successfully used for a similar purpose. After the administration of the remedy, and when the stomach is calmed, the usual remedies are resorted to. The substances ejected from the stomach, in these cases, consist, for the most part, of vitiated bile, the quantity being at times exceedingly great. When the bowels have at length given way to the effects of medicine, at times, enormous quantities of feculent matter, with flatus, are discharged, and if not moderated, the catharsis would endanger the life of the patient.

"For this purpose, if pain still remains, I give castor oil and laudanum, or, if the stomach reject this, small doses of spirits of turpentine and olive oil, alternated with laudanum. But should torpor of the bowels still remain after the violence of the disease has been overcome, I have found a useful remedy in a mixture of castor oil and spirits of turpentine, in the proportion of a table-spoonful of the former, to a tea-spoonful of the latter. In more obstinate cases of torpidity of the bowels, under these circumstances, I have resorted to the use of croton oil, with signal advantage. This was particularly the case in two patients, who, after recovery from a severe attack of colica pictonum, had a *relapse,* without any subsequent exposure to white lead—having been seized with catarrh from exposure to cold and damp." 578.

The cases of paralysis of the extremities, usually of the fore-arm, which he has witnessed, have, with the exception of one, done well under the use of powdered nux vomica, caustic issues on each side of the nape of the neck, and purging medicines, continued for two or three months.

Notwithstanding, continues our author, the utmost care and attention on the part of the medical attendant, this disease will frequently terminate in inflammation of the intestines; but this is by no means necessarily fatal, provided the attention be attracted in time to this symptom. Post-mortem examinations, at least such as occurred under his own inspection, have invariably displayed mortification of the intestines, provided mania à potu had not supervened and carried off the patient. Four cases only have terminated fatally under his own treatment. Three of these were habitually intemperate; and the other, an ignorant German, unacquainted with the dangerous nature of his complaint, suffered himself to be neglected for three days previous to making application for relief.

From the severity of the disease, together with the use of the necessarily violent remedies, dysentery, with copious discharges of blood, is not unfrequently induced; in which case, no remedy is more effectual in relieving pain and suppressing the discharge, than the pulverized saccharum saturni, in combination with calomel, opium, and ipecacuanha. Should the blood discharged, after long-continued constipation, present a grumous or coffee-ground appearance, it indicates commencing mortification, which, for the most part, speedily ends in the death of the patient.

### 6. *Remarkable Case of Intussusception.*

On the 1st of May, 1819, Dr. H. was called to a child, aged 5 months; he found her very restless, with a tongue white and furred, little or no fever, and such irritability of stomach that she had rejected the greater part of the medicines which had been given to her. Dr. H. prescribed calomel, castor-oil, and a turpentine enema. The irritability of the stomach ceased so far as to allow of these remedies being retained, but no action could be obtained from the bowels, and attention being directed to the fundament, there was observed what Dr. H. took to be an inversion of the rectum; which protruded nearly an inch beyond the verge of the anus every time the patient strained. At 12 p.m. the little patient died.

*Dissection.*—On opening the abdomen the dissectors were struck with the size and position of the colon, which appeared as if distended with fæces of a black colour, extending no further than the left hypochondriac region, where an intussusception was evident.

They found the intussusception commence in that portion of ileum just before it enters the caput coli, but instead of entering, as usual, in the right iliac, it was found in the left hypochondriac region.

The parts involved in the disease were as follow: about one inch of the ileum, the whole of the cœcum, with a portion of the colon, were received into the remaining portion of the colon; the intussuscepted portions of the cœcum and colon were of course inverted. The cœcum had descended into the rectum; which gave to the latter the appearance of a bowel distended with hardened fæces, whereas there was not more than half an ounce of fæcal matter in the whole extent of the colon, and that was of a fluid consistence.

The cœcum was thickened from inflammation; and its inner surface, together with a portion of strictured colon, was in a state of gangrene. It was this hardened, tumefied, and inverted cœcum which had descended beyond the verge of the anus during the act of straining, and which Dr. H. had mistaken for prolapsus of the rectum.

"Had the precise nature of this case been known, it would have been impossible to have overcome the obstruction by any mechanical means, as, after the parts were taken out of the body, we were unable to dissolve their connexions without laceration—after such separation, however, we were enabled to produce an artificial intussusception precisely similar to the original." 565.

### 7. *Some Experiments and Observations on the Poison of the Rattlesnake.*

Perhaps we may be excused for observing that very erroneous ideas are entertained by many medical men on the subject of animal poisons. Their effects are singularly modified by the state of the individual who is their recipient, and the same poison, in the same dose, which will produce serious symptoms in one person, may occasion very trivial ones in another, and no perceptible consequences in a third. It is to this circumstance that we may mainly attribute the reputed efficacy of many of those antidotes that swell the measure of popular, we might almost add of professional credulity.

Some observations and experiments of Dr. Harlan's on the poison of the rattlesnake seem to us so well calculated to illustrate an important practical principle, that we shall briefly notice them.

A collection of rattlesnakes was exhibited in Philadelphia, by Messrs. Elnsworth and Murray. They professed to be in possession of a specific against the effects of the bite, and handled the animals with the utmost fearlessness. They even offered to submit to being bitten for the sake of the experiment, and a committee, including Dr. Harlan, witnessed and superintended it.

On the 15th of December, 1827, Mr. Elnsworth, at 12 h. 15 m. exposed his left hand to a large active male snake,* purposely irritated, and received a wound from a single fang on the back of his hand, directly over a prominent venous branch. A large drop of transparent, yellowish, and glairy

* He had previously received a bite from a female snake.

fluid was spread over and around the wound, which was doubtless ejected from the poison sack. A little very dark blood slowly exuded from the wound.

12 h. 31 m. Slight swelling is observable immediately around the second bite.

12 h. 48 m. Elnsworth again exposed his hand to the female snake, and received two additional punctures simultaneously, one from each fang, on the lower extremity of the metacarpal bone of the ring-finger. As in the first instance, neither of these wounds displayed symptoms of the specific effects of the poison; the *second bite* therefore, or that received from the male snake, will alone be the subject of further observations in this experiment.

1 o'clock, p.m. The swelling around the second bite has increased considerably, the tumefaction extending up and down along the course of the vein, about an inch and a half in length, and half that size in breadth, the greatest length of the tumefaction being below the wound. The man now complained of pain and numbness along the course of the lymphatic vessels on the inner part of the fore-arm.

1 h. 25 m. Pulse natural, symptoms last described somewhat increased; swelling unattended with symptoms of inflammation.

1 h. 30 m. Although the man is perfectly willing to permit the symptoms to proceed further, several of the witnesses expressed their unwillingness to bear the responsibility of the consequences; he was therefore permitted to have recourse to his remedy, and he immediately swallowed a few ounces of the decoction of the root, and appeared indifferent about the external application of the same to the wound. He stated that the original stock of the vegetable being exhausted, and the season too far advanced to enable him to obtain more at present, he would be under the necessity of applying portions of the flesh of one of the reptiles (just decapitated for the purpose of another experiment) to the wound.

2 h. 30 m. He has held the bloody portion of the snake to his wound incessantly, from which all the swelling has subsided, together with all uneasy sensations, from his hand and arm.

4 o'clock, p. m. The man Elnsworth has remained constantly in the room under inspection. His dinner was offered to him, but he had little disposition for food; says his stomach is a little sick, probably the effects of the medicine. No tumefaction or other symptoms remain; the wounds resemble slight scratches, without any appearance of inflammation. The vein in which the bite took effect presents a peculiar appearance, being for the distance of an inch between the valves above and below the wound quite empty. Directly above the valve the vein is unusually prominent, and the pressure, from the application of the flesh, has been removed for more than an hour.

The root proved on inspection to be that of the *Hieraceum venosum*, or Hawk-weed, Adder's-tongue, Poor Robin's plantain, Rattlesnake weed, &c. a common weed in the dry open woodlands.

The man, therefore, experienced few or no bad effects from his bites, whether by the female or the male snake. It was an object to determine whether animals would suffer from them.

*Experiment.*—11 h. 31 m. A pup, about three or four weeks old, was bitten by the same female snake which had previously bitten Elnsworth in the first experiment: both fangs took effect, and the two wounds were about an inch and a quarter apart.

11h. 34m. Pup urinates.

11h. 36m. Cries and staggers.

11h. 37m. Belly tense in the vicinity of the wound, and apparently painful; the wound presents an ecchymosis, being tumid and of a dark colour.

11h. 39m. Pup lies on his side, and continues his plaintive cries, also emits some froth from the mouth. The ecchymosis increases rapidly, and a pale bloody humour exudes from the wounds.

11h. 51m. The animal is quiet and fainting.

12 o'clock, merid. Appears vertiginous, turning round and resting on his extended fore-feet; staggering and resting on his side, and turning upon his back. These symptoms continued with little alteration until

4 o'clock, When the animal died, having previously exhibited some stertorous breathing, but without the occurrence of convulsions.

Was the recovery of Elnsworth due to the remedy, or to the natural powers?

*a.* Five experiments were made on animals (inclusive of the last) to determine whether death would necessarily ensue, if the creatures were left to the efforts of Nature. We shall quote one.

*Experiment.*—4, p.m. A stout pup was inoculated with the poison, expressed from the poison-bag of a living snake, on the left side of the abdomen.

4h. 15m. Local symptoms are evident, and constitutional effects are beginning to be manifested.

5 o'clock, p.m. Symptoms much increased; the animal cries with pain and uneasiness; changes its posture frequently; moves with a tottering and irregular gait, sometimes lying on its breast with the fore feet extended; these symptoms were occasionally interrupted with drowsiness, and finally the animal went into a deep sleep.

9 o'clock, p.m. The pup commenced licking his wound, the swelling of which, from the ecchymosis, had so increased as to hang down like a large hernia.

The succeeding day this animal also recovered, no symptom remaining except a slight tenderness in the part where the inoculation had been performed.

Dr. Harlan adds:—

"Of all the animals bitten in these experiments, one only died, though all were more or less affected by the poison. Although the wound which was inflicted on Mr. Elnsworth was attended with the usual *local* effects, there is no proof that the poison would have proved mortal without the use of the remedy, inasmuch as obvious local effects were observed in some of the animals that finally recovered without the interference of art. Though at the same time it will be remembered that the first animal experimented on died from the poison of the same snake which had previously inflicted a wound on the man." 504.

*b.* Some, that we might term crucial experiments, were instituted to ascertain the powers of the antidote. The experiments were three in number. We shall quote the second.

*Experiment.* At 4h. 21m. a small black pup was bitten by an active male snake.

At 4h. 34m. a brown dog was bitten by the same snake severely in the foot; the wound bled freely.

At 4h. 37m. the black dog was again bitten in the foot, the wound being severe.

At 4h. 40m. black dog was drowsy, and unable to stand.

At 4h. 45m. brown dog evacuated per anum.

At 4h. 46m. black dog evacuated per anum.

At 4h. 47m. administered a quarter of a pint of the decoction to the black dog.

At 4h. 55m. gave the same dog more of the decoction, say half a pint in all; he is certainly not more drowsy, while the brown dog appears very sick and restless; the black dog swelled a great deal, but shows signs of more liveliness.

At 5h. 25m. gave the black dog half a wine-glassful more; he trembles very much, and the leg is greatly swelled, but he swallows his medicine easily.

5h. 45m. black dog drinks of the decoction voluntarily, and at 6h. 30m. went to sleep. The brown dog has become more lively, and limps about the room; the parts in the vicinity of the wounds of both are much tumefied. About this period both became considerably revived; bloody serum was squeezed out of the black pup's wound, and the swelling thus diminished. On the following morning the black dog was found dead, whilst the brown dog recovered completely.

We think these points completely established:—*that* the poison of the rattlesnake may prove fatal to an animal—*that* the poison may produce local symptoms of more or less severity, and that the animal may recover without the interference of art—*that* the poison may produce no general and few or no local symptoms—*that* the hieraceum venosum is *not* a specific against the poison.

These experiments have a wider interest and a more extensive application than may, at first sight, be apparent. Mutatis mutandis, what is proved of the hieraceum may apply to the eau-de-luce, &c. They tend to encourage that rational scepticism so beneficial to science, for here were persons of great empirical experience, exhibitors of rattlesnakes, fearlessly exposing themselves to be bitten on the strength of the virtues of a drug, which, tested by the rational experiments of a physician, could not save the life of a puppy. And such empirical faith may be seen in more professional people than showmen, and walks abroad under the doctor's, nay the lecturer's garb.

